# Branchiomeric Muscle Development Requires Proper Retinoic Acid Signaling

**DOI:** 10.3389/fcell.2021.596838

**Published:** 2021-07-09

**Authors:** Qi Wang, Lin Xu, Jiro Miura, Mithun Kumar Saha, Yume Uemura, Lisa L. Sandell, Paul A. Trainor, Takashi Yamashiro, Hiroshi Kurosaka

**Affiliations:** ^1^Department of Orthodontics and Dentofacial Orthopedics, Graduate School of Dentistry, Osaka University, Suita, Japan; ^2^The Affiliated Stomatology Hospital, Zhejiang University School of Medicine, Hangzhou, China; ^3^Key Laboratory of Oral Biomedical Research of Zhejiang Province, Zhejiang University School of Stomatology, Hangzhou, China; ^4^Division for Interdisciplinary Dentistry, Osaka University Graduate School of Dentistry, Suita, Japan; ^5^Department of Oral Immunology and Infectious Diseases, University of Louisville School of Dentistry, Louisville, KY, United States; ^6^Stowers Institute for Medical Research, Kansas City, MO, United States; ^7^Department of Anatomy and Cell Biology, University of Kansas Medical Center, Kansas City, KS, United States

**Keywords:** cranial muscle development, retinoic acid signaling, craniofacial abnormalities, muscle progenitor cell, muscle differentiation

## Abstract

The first and second branchiomeric (branchial arch) muscles are craniofacial muscles that derive from branchial arch mesoderm. In mammals, this set of muscles is indispensable for jaw movement and facial expression. Defects during embryonic development that result in congenital partial absence of these muscles can have significant impact on patients’ quality of life. However, the detailed molecular and cellular mechanisms that regulate branchiomeric muscle development remains poorly understood. Herein we investigated the role of retinoic acid (RA) signaling in developing branchiomeric muscles using mice as a model. We administered all-trans RA (25 mg/kg body weight) to Institute of Cancer Research (ICR) pregnant mice by gastric intubation from E8.5 to E10.5. In their embryos at E13.5, we found that muscles derived from the first branchial arch (temporalis, masseter) and second branchial arch (frontalis, orbicularis oculi) were severely affected or undetectable, while other craniofacial muscles were hypoplastic. We detected elevated cell death in the branchial arch mesoderm cells in RA-treated embryos, suggesting that excessive RA signaling reduces the survival of precursor cells of branchiomeric muscles, resulting in the development of hypoplastic craniofacial muscles. In order to uncover the signaling pathway(s) underlying this etiology, we focused on *Pitx2*, *Tbx1*, and *MyoD1*, which are critical for cranial muscle development. Noticeably reduced expression of all these genes was detected in the first and second branchial arch of RA-treated embryos. Moreover, elevated RA signaling resulted in a reduction in *Dlx5* and *Dlx6* expression in cranial neural crest cells (CNCCs), which disturbed their interactions with branchiomeric mesoderm cells. Altogether, we discovered that embryonic craniofacial muscle defects caused by excessive RA signaling were associated with the downregulation of *Pitx2*, *Tbx1*, *MyoD1*, and *Dlx5/*6, and reduced survival of cranial myogenic precursor cells.

## Introduction

Craniofacial muscles comprise two groups: (1) extraocular muscles, which control eye movement which derive from cranial mesoderm, and (2) branchiomeric muscles, which derive from branchial arch mesoderm. The first branchial arch (BA1, dorsal/rostral maxillary process and a ventral/caudal mandibular process) mesoderm gives rise to progenitors of jaw muscles, including the temporalis, pterygoid, masseter, mylohyoid and anterior digastric; the second branchial arch (BA2, hyoid arch) mesoderm gives rise to progenitors of facial expression muscles, including the auricularis, buccinator, posterior digastric, frontalis, orbicularis oculi, quadratus labii, and zygomaticus; and other caudal BA-derived muscles are associated with laryngeal and pharyngeal muscles (Noden, 1983, 1986; Trainor et al., 1994; Lescroart et al., 2010). Other muscles within the head such as the intrinsic and extrinsic muscles of the tongue derive from occipital somites, whose myogenic precursors migrate as the hypoglossal cord (Noden, 1983; Huang et al., 1999; Noden and Francis-West, 2006; Parada et al., 2012).

Branchiomeric muscles have a gene regulatory program distinct from that of the trunk muscles. For example, *Pitx2* and *Tbx1* are important upstream regulators of skeletal myogenesis in the branchial arches but not myogenesis in the trunk (Grifone and Kelly, 2007). *Pitx2* is a paired-related homeobox gene that regulates transcription of the myogenic regulatory factor genes (*MRFs*) as well as genes encoding essential factors for proliferation and survival of muscle progenitors in the branchial arches (Dong et al., 2006). Although *Pitx2* is expressed in the mesodermal cores of all BAs when myoblasts can be detected, its expression is required to establish premyoblast specification only in BA1 (Shih et al., 2007). *Tbx1*, which is expressed in the premyoblast mesoderm in BA1 and BA2, collaborates with *Pitx2* as part of the core myogenic program to generate head muscles. Additionally, these genes are known to collaborate with other core myogenic program factors to generate head muscles (Dong et al., 2006; Dastjerdi et al., 2007). Similarly, *Tbx1* is required for activating transcription of *MRFs*, such as *Myf5* and *MyoD*, at the onset of myogenic commitment in branchial mesoderm (Kelly et al., 2004). Moreover, *Tbx1* mutant mice present with sporadic failure of development of muscles that normally originate from BAs (Jerome and Papaioannou, 2001; Dastjerdi et al., 2007; Grifone et al., 2008).

Cranial neural crest cells (CNCCs) are another important cell population which populate the BAs together with mesodermal cells (Trainor et al., 1994; Trainor and Tam, 1995; Chai et al., 2000). Via their interactions with mesodermal cells, CNCCs direct branchiomeric muscle development (Noden, 1983, 1986; Heude et al., 2010; Ziermann et al., 2018). Distal-less homeobox (DLX) proteins provide CNCCs with patterning information and intra-arch polarity along the dorsoventral/proximodistal axis (Heude et al., 2010; Minoux and Rijli, 2010). *Dlx5* and *Dlx6* are expressed in CNCCs of the mandibular process, and are necessary for CNCCs-mesoderm interactions during craniofacial myogenesis, since inactivation of *Dlx5* and *Dlx6* results in the loss of jaw muscles and compromised tongue development (Heude et al., 2010).

Previous studies have shown that RA signaling is essential for ocular, jaw and branchial muscle development (Bothe et al., 2011; Bohnsack et al., 2012; Rhinn and Dolle, 2012; Kurosaka et al., 2017; Wang et al., 2019). Retinoic acid (RA) is derived from liposoluble vitamin A (retinol) and *in vitro*, low concentrations of RA enhance skeletal myogenesis in stem cells and myoblast cell lines by regulating muscle progenitor factors and/or MRF expression (Edwards and McBurney, 1983; Albagli-Curiel et al., 1993; Halevy and Lerman, 1993; Kennedy et al., 2009). During heart development, RA signaling is required for ventricular myocyte proliferation (Lavine and Ornitz, 2008; Nakajima, 2019). However, the function of RA signaling *in vivo* during craniofacial muscle development is poorly understood.

In this study, we showed that maternal RA-exposure resulted in malformation of branchiomeric muscles derived from BA1 and BA2. This phenotype was associated with elevated cell death of branchial mesodermal cells, from which branchiomeric muscles arise. Additionally, excessive RA signaling resulted in reduced expression of *Pitx2* and *Tbx1*, which underpinned the myogenic specification and determination defects in BA1 and BA2. Moreover, excessive RA signaling repressed *Dlx5* and *Dlx6* expression in CNCCs in the proximal region of the BAs, which disturbed CNCCs-mesoderm interactions during branchiomeric muscle development. Taken together, our results have revealed novel molecular and cellular mechanisms linking elevated RA signaling and branchiomeric muscle malformation.

## Results

### Excessive RA Signaling Result in BA1- and BA2-Derived Muscle Malformations

**FIGURE 1:**
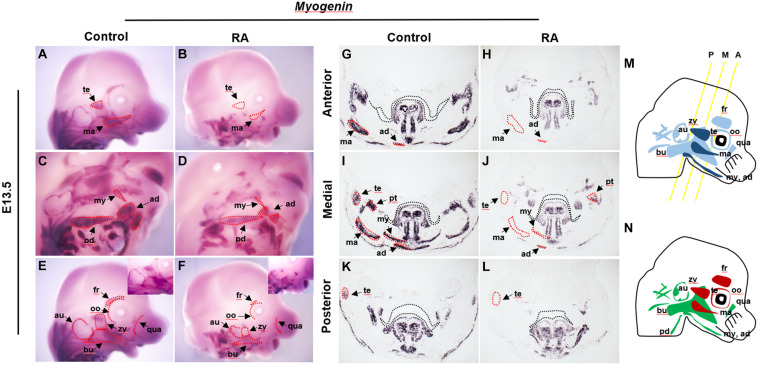
Abnormalities of BA1 and BA2 derived muscles in RA-treated embryos. *Myogenin in situ* hybridization for whole-mount **(A–F)** and frontal sections from anterior **(G,H)**, medial **(I,J)**, and posterior **(K,L)** regions (see M for approximate levels of sections along the anterior-posterior axis) of E13.5 control **(A,C,E,G,I,K)** and RA-treated **(B,D,F,H,J,L)** embryos. In **(G–L)** black dashed lines indicate the margin of palatal shelf and tongue primordium. In RA-treated embryos, the BA1-derived temporalis (te) and masseter (ma) muscles are absent (marked by red dashed lines in **B,H,J,L**); pterygoid (pt), mylohyoid (my) and anterior digastric (ad) muscles are reduced to small components (compare in **D,H,J,L** to **C,G,I,K**). **(C–F)** The RA-treated BA2-derived muscles are either absent (red dashed line) or replaced by a few muscle fibers. Higher magnifications are shown in upper right boxes of **(E,F)**. **(M,N)** Schematics of BA1- and BA2-derived muscles. **(M)** Dark blue indicates muscles of BA1 origin; light blue indicates muscles of BA2 origin. Yellow lines illustrate the levels of frontal sections shown in **(G–L)**. A, anterior; M, medial; P, posterior. **(N)** Red indicates absent in RA-treated embryos; green indicates reduced in RA-treated embryos. ad, anterior digastric muscle; au, auricularis muscle; bu, buccinators muscle; ma, masseter muscle; my, mylohyoid muscle; oo, orbicularis oculi muscle; pd, posterior digastric muscle; pt, pterygoid muscle; qua, quadratus labii muscle; te, temporalis muscle; zy, zygomaticus muscle.

The first and second branchial arch mesoderm cells contribute to the jaw muscles – the temporalis, pterygoid, masseter, mylohyoid and anterior digastric, and to the facial expression muscles – the auricularis, buccinator, posterior digastric, frontalis, orbicularis oculi, quadratus labii, zygomaticus and others, respectively (Figure 1M) (Lescroart et al., 2010). To examine the effect of exogenous RA signaling on branchiomeric muscle development, we performed *in situ* hybridization using *Myogenin* as a myogenic determination marker (Hasty et al., 1993) in heads of E13.5 RA-treated embryos (Figures 1A–L). Muscles derived from BA1 displayed specific and consistent abnormalities in association with elevated RA signaling. The temporalis and masseter muscles failed to form (Figures 1B,H,J,L), and the pterygoid, mylohyoid and anterior digastric muscles were present, but were reduced in size and thickness, and the buccinator muscles were fragmented, as evidenced by the expression of *Myogenin* (Figures 1D,H,J,L). In the case of muscles derived from BA2, the frontalis and orbicularis oculi muscles were missing, whereas the others were hypoplastic and disorganized compared to controls (Figures 1D,F). The phenotypes of individual muscles derived from BA1 and BA2 are summarized in Figure 1N. Both intrinsic and extrinsic tongue muscles which originate from the occipital somite (Noden and Francis-West, 2006), formed normally and did not show any noticeable structural abnormality in RA-treated heads at E13.5 (Figures 1G–L and Supplementary Figure 1). Together, these data showed that excessive RA signaling results in defects in the development of BA1- and BA2-derived cranial muscles. In order to understand the cellular mechanism underlying this phenotype, we further analyzed the myogenic developmental process in RA-treated embryos.

Conversely, we also assessed the effect of reduced RA signaling on developing craniofacial muscle using conditional *Rdh10* knock out embryos (*CreErt2;Rdh10^*flox/flox*^*), which exhibit a severe reduction of RA signaling in the craniofacial region (Kurosaka et al., 2017). Interestingly, most of the cranial muscles showed subtle differences between control and *CreErt2;Rdh10^*flox/flox*^* embryos (Supplementary Figure 2).

### Elevated RA Signaling Affects Myogenic Gene Expression During Myogenesis in BA1 and BA2

**FIGURE 2:**
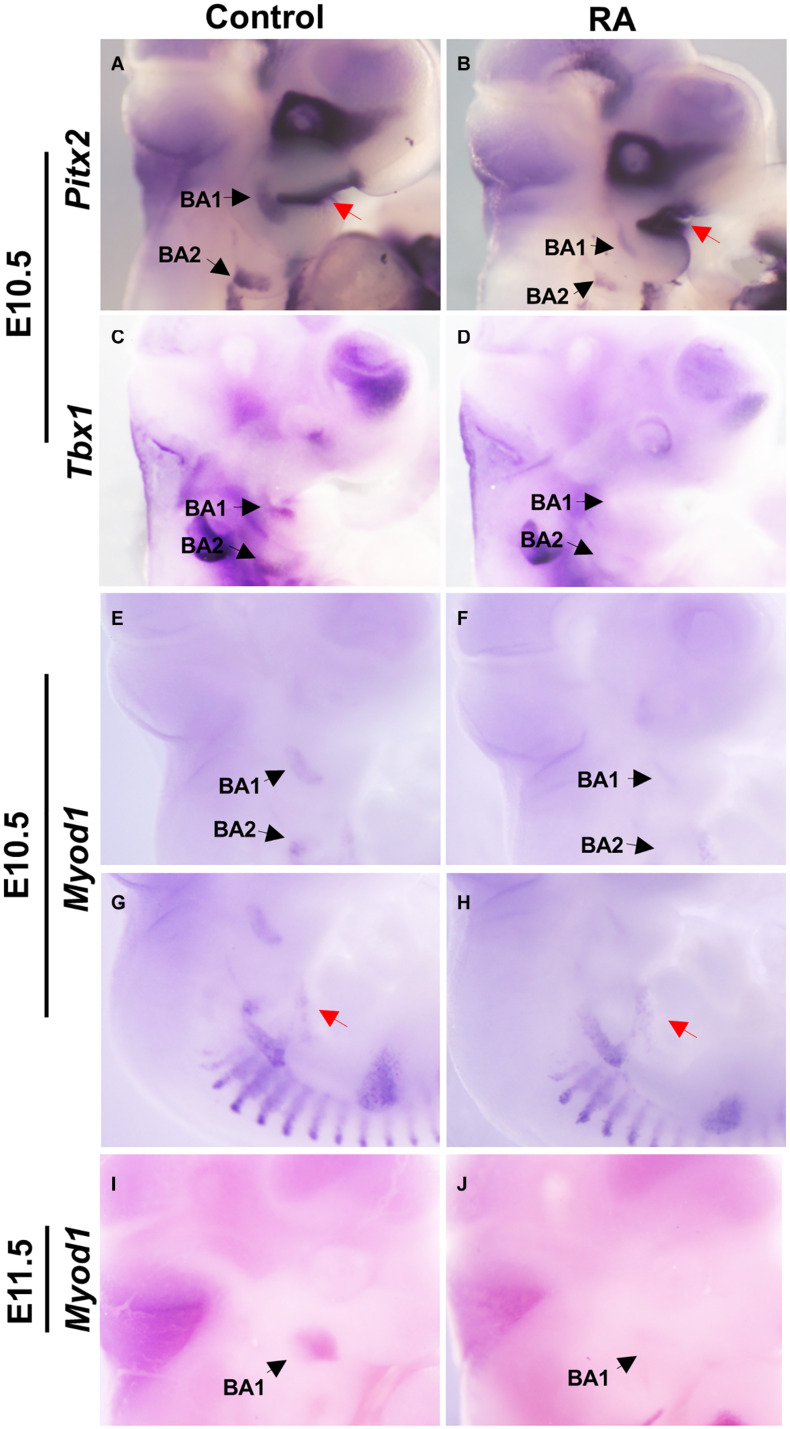
Disruption of early myogenic markers in BA1 and BA2 of RA-treated embryos. Lateral views of whole-mount *in situ* hybridization for *Pitx2* **(A,B)**, *Tbx1* **(C,D)**, and *MyoD1* **(E–J)** in E10.5-E11.5 BA1 and BA2 of control **(A,C,E,G,I)** and RA-treated **(B,D,F,H,J)** embryos. In BA1 and BA2 at E10.5, the expression of the early markers of myogenic specification, *Pitx2* **(A,B)** and *Tbx1* **(C,D)**, is severely affected by the RA treatment. However, the expression of *Pitx2* is higher in the oral ectoderm in RA-treated embryos (**A,B**, red arrows). **(E–J)** *MyoD1*, a marker of myogenic determination, fails to be expressed in the BA1 and BA2 in embryos treated with RA at E10.5 and E11.5, but no significant change is observed in the hypoglossal cord (**E,F**, red arrows). Black arrows denote BA1 and BA2 hybridization signal or absence of signal.

We examined the expression patterns of *Pitx2* and *Tbx1*, which mark muscle precursor cells in BA1 and BA2. In the control embryos, *Pitx2* was expressed at E10.5 in the mesodermal core of BA1 and BA2, while in the RA-treated embryos *Pitx2* expression was substantially reduced (Figures 2A,B). In contrast, increased *Pitx2* expression could be observed in the epithelium of BA1 (Figures 2A,B). *Tbx1* expression was also reduced in BA1 and BA2 of E10.5 RA-treated embryos compared to controls (Figures 2C,D). These results indicated that excessive RA signaling interferes with the expression of *Pitx2* and *Tbx1*, which are essential for differentiation of BA1 and BA2 myogenic mesoderm.

The myogenic determination factor *MyoD1* is under the control of both *Pitx2* and *Tbx1* (Braun and Gautel, 2011). In E10.5 control embryos, *MyoD1* was expressed in BA1 and BA2 myoblasts, which later develop into the masticatory and facial premuscle masses at E11.5. In RA-treated embryos, *MyoD1* expression was a substantially reduced in BA1 and BA2, which is indicative of a defect in myogenic determination (and later differentiation). Furthermore, this is likely an effect of perturbed specification due to reduced *Pitx2* and *Tbx1* expression in BA1 and BA2 (Figures 2E,F,I,J). In contrast, *MyoD1* expression in the hypoglossal cord, from which the tongue myoblasts migrate (Huang et al., 1999), remained unchanged in the RA-treated group (Figures 2G,H).

Collectively, these findings indicate that excessive RA perturbs BA1 and BA2 muscle precursor specification and determination. We also analyzed the expression of *Aldh1a2* and *Aldh1a3*, which encode indispensable enzymes for synthesizing RA, in RA-treated and RA loss-of-function, *CreErt2;Rdh10^*flox/flox*^* embryos. We detected subtle differences in the expression of these genes in the developing craniofacial region, between control and RA-treated embryos. This was also true for the expression of *Aldh1a2* in *Rdh10* mutant embryos, whereas in contrast *Aldh1a3* expression was substantially reduced in the head (Supplementary Figure 3).

### Increased Apoptosis of the Muscle Progenitor Cells in BA1 and BA2 Contributes to RA-Induced Branchiomeric Muscle Defects

**FIGURE 3:**
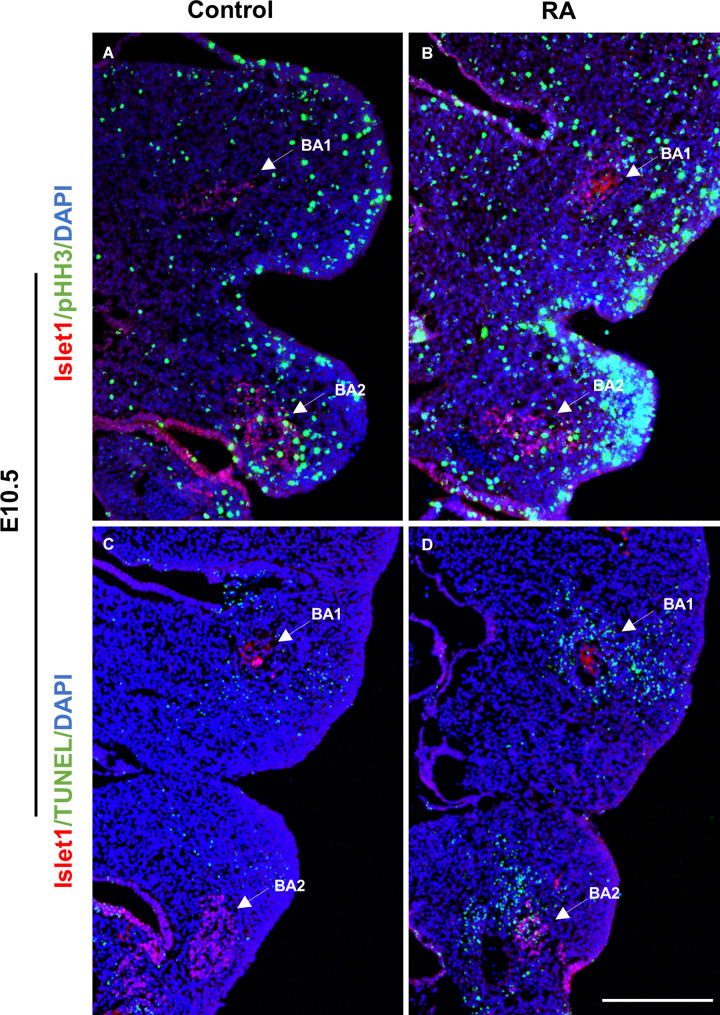
Unaltered proliferation but increased apoptosis in the muscle progenitor cells of the BA1 and BA2 at E10.5 after RA treatment. Immunofluorescent detection of pHH3 (green; **A,B**) and TUNEL (green; **C,D**) in transverse sections of control **(A,C)** and RA-treated **(B,D)** E10.5 embryos. Arrows indicate Islet1-positive (red) muscle progenitor cells in the BA1 and BA2 regions. Scale bar: 250 μm.

*Pitx2* and *Tbx1* have previously been implicated in proliferation and survival of muscle progenitor cells in the branchiomeric muscles (Kelly et al., 2004; Dong et al., 2006; Shih et al., 2007) and we observed altered *Pitx2* and *Tbx1* expression in E10.5 RA-treated embryos. We further investigated the behavior of BA1 and BA2 muscle progenitor cells via staining for Islet1 (Nathan et al., 2008) in conjunction with phosphorylated Histone H3 (pHH3) and TUNEL to assess cell proliferation and cell death, respectively. Cell proliferation was unchanged in the muscle progenitor cells of both BA1 and BA2 in E10.5 RA-treated embryos compared to controls (Figures 3A,B and Table 1). In contrast, increased cell death was detected in the muscle progenitor cells of both BA1 and BA2 in E10.5 RA-treated embryos compared to controls (Figures 3C,D and Table 1). These results demonstrate that excessive RA signaling results in cell death in muscle progenitor cells in both BA1 and BA2.

**TABLE 1:** Cell proliferation and cell death in ISLET1 positive cells in the developing branchial arches (BA).

		Control group	RA group	OR (95%CI)	*P*-value
No of PHH(+) cells/Total No of cells in the field	BA1	23/122 (19%)	29/155 (18%)	1.00 (0.52–1.93)	1.00
	BA2	53/362 (15%)	69/405 (17%)	0.83 (0.55–1.25)	0.37
No of TUNEL(+) cells/Total No of cells in the field	BA1	2/152 (1%)	43/169 (25%)	0.03 (0.004–0.15)	*0.004
	BA2	3/467 (1%)	77/445 (17%)	0.03 (0.006– 0.09)	*0.006

*OR, Odds ratio; CI, Confidence interval. ^∗^p < 0.05.*

### Abnormalities of BA1-Derived Jaws Are Associated With *Dlx5* and *Dlx6* Expression Defects in RA-Treated Embryos

**FIGURE 4:**
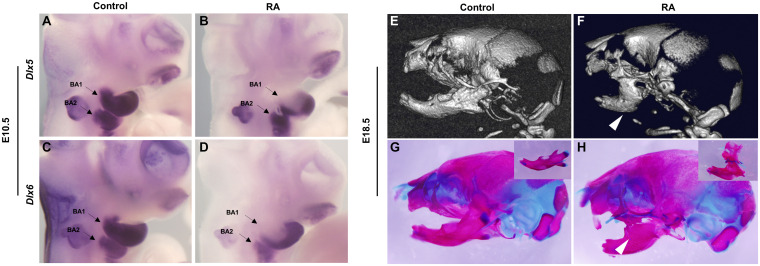
Abnormalities of BA1-derived jaws and associated *Dlx5* and *Dlx6* expression defects in RA-treated embryos. Lateral views of whole-mount *in situ* hybridization for *Dlx5* **(A,B)** and *Dlx6* **(C,D)** in E10.5 BA1 and BA2 of control **(A,C)** and RA-treated **(B,D)** embryos. **(B,D)** The expression of *Dlx5* and *Dlx6* in BA1 is lost in proximal but not in distal regions of RA-treated embryos at E10.5. In BA2, the expression of Dlx5 and Dlx6 is not affected in E10.5 RA-treated embryos. Arrows denote BA1 and BA2 hybridization signal or absence of signal. Micro-CT scans **(E,F)** and Alizarin red (bone) and Alcian blue (cartilage) stained skeletal preparations **(G,H)** of E18.5 control **(E,G)** and RA- treated **(F,H)** embryos. The white arrowheads in **(F,H)** mark the syngnathia in RA- treated embryos. Higher magnifications of dissected jaws are shown in the boxes of **(G,H)**.

CNCC-mesoderm interactions are crucial for proper branchiomeric myogenesis (Grenier et al., 2009) and we have previously shown that excessive RA signaling at E8.5 affects CNCC development (Wang et al., 2019). Therefore, we hypothesized that a defect in CNCC development could underly the etiology of RA-induced BA1- and BA2- derived muscle malformation. To test this idea, we assessed the activity of *Dlx5* and *Dlx6*, which are expressed by CNCCs and are required for the myogenic differentiation and patterning of craniofacial muscles (Heude et al., 2010). *Dlx5* and *Dlx6* expression was down-regulated in the proximal but not regions of distal branchial arches in E10.5 RA-treated embryos. The reduction was more pronounced in BA1 than BA2 (Figures 4A–D). Therefore, the BA1-derived muscle anomalies observed in the RA-treated embryos likely manifest as a result of perturbed *Pitx2* and *Tbx1* expression in the progenitor branchial arch mesoderm, together with loss of *Dlx5* and *Dlx6* expression by CNCCs in the proximal regions BA1, which impacts proximal-distal patterning. We also detected bilateral fusion of the upper and lower jaw in RA-treated embryos (Figures 4E–H). To evaluate the phenotype in detail, we dissected the mandibular structures from skeletal preparations of E18.5 embryos. In the control embryos, the main features – the coronoid process, the condylar process, the angular process and the molar alveolus – could be recognized (Figure 4G, box). In contrast to the controls, the position around the coronoid process and dentary bone in the mandible of RA-treated embryos was fused to the posterior/lateral position of maxilla (Figure 4H, box). Additionally, the condylar process was not detectable and the angular process was reduced in size (Figure 4H, box). The altered expression of *Dlx5* and *Dlx6* in the BAs could also have contributed to causing these defects (Vieux-Rochas et al., 2007; Vieux-Rochas et al., 2010).

### The Expression Pattern of Retinoic Acid Receptors in Developing Craniofacial Region With Elevated or Reduced RA Signaling

RA signaling is mediated by a nuclear receptor superfamily consisting of multiple RARs (RARα, RARβ, and RARγ) and their heterodimeric binding partner RXRs (RXRα, RXRβ, and RXRγ). In order to further analyze the effects of enhanced and reduced RA signaling during craniofacial muscle development, the expression of *RARs* and *RXRs* was analyzed in both RA-treated and *CreErt2;Rdh10^*flox/flox*^* embryos whose dams were administered tamoxifen at E7.0. Overall, we detected subtle differences in the expression levels and patterns of both *RARs* and *RXRs* between the control group and RA-treated embryos at E11.0 except that *Rxra* showed reduced expression in the developing frontonasal process (Supplementary Figure 4). In contrast, the expression of *RARa*, *RARb*, *RXRa*, *RXRb*, and *RXRg* was substantially reduced in the developing craniofacial region of E11.5 *CreErt2;Rdh10^*flox/flox*^* embryos compared to controls(Supplementary Figure 4).

## Discussion

The first and second branchiomeric (branchial arch) muscles form part of the craniofacial musculature. Abnormal development of these muscles during embryogenesis results in congenital defects in the muscles of mastication and facial expression. Surgical reconstruction of malformed branchiomeric muscles is complex, often requires multiple procedures, and is not routinely available in many healthcare systems. Therefore, understanding the detailed molecular and cellular mechanisms regulating branchiomeric muscle development is critical to our understanding of the etiology of congenital muscle defects and to develop possible therapies. Previous reports have shown that RA signaling is essential for ocular and BA development in the craniofacial region (Sandell et al., 2007; Bohnsack et al., 2012; Rhinn and Dolle, 2012). In this study, we demonstrated that excessive embryonic RA signaling causes branchiomeric muscle malformations due to defects in myogenic specification in BA1 and BA2. This phenotype was associated with elevated apoptosis of muscle progenitor cells in BA1 and BA2. Furthermore, RA-induced proximal loss of *Dlx5* and *Dlx6* expression disturbs critical cellular interactions between CNCCs and muscle precursor cells during branchiomeric muscle development (Heude et al., 2010).

### Diverse Reactions of Muscle Precursor Cells to RA Signaling

Numerous studies have attempted to elucidate the effects of stage- and/or dose- dependent effects of RA signaling on the development of various different muscles. It has been reported that RA signaling exhibits different effects on the differentiation of embryonic stem cells into cardiomyocytes depending on the timing of RA supplementation (Edwards and McBurney, 1983; Wobus et al., 1997; Nakajima, 2019). Compared with the levels of RA that enhance cardiomyogenesis, lower levels of RA signaling are known to enhance skeletal myogenesis (Edwards and McBurney, 1983; Halevy and Lerman, 1993). Previous studies have shown that both facial and tongue muscles are severely affected after RA-treatment (200 mg/kg) at E8.0 in the mouse fetus (Padmanabhan and Ahmed, 1997). We examined the effects of dose and timing of RA administration in our previous work on the effect of exaggerated RA signaling midgestational development. As a result, we found that daily administration of 25 mg/kg RA from E8.5 to E10.5 had the effect of disrupting multiple craniofacial structures (Wang et al., 2019). Also, in the present study, we detected BA1- and BA2-derived muscle malformations but minimal effects of RA-treatment on tongue muscle development. Our results indicate that proper RA signaling is essential for survival of muscle precursor cells in the branchial arch mesoderm. Branchiomeric muscle development is mediated by signaling and genetic pathways that are distinct from those of extraocular, tongue and laryngeal muscles in the head (Braun and Gautel, 2011). Interestingly, our study showed that in contrast to the induction of branchiomeric muscle defects, excess RA signaling did not cause defects in *MyoD1* expression in the hypoglossal cord before E13.5. Altogether, these results indicate that muscle precursor cells react to RA signaling in different ways depending on the tissue type, developmental timing and dosage of RA. Interestingly, reduced RA signaling did not show as strong a disruption of BA1- and BA2-derived muscle development as elevated RA signaling. These results indicate that craniofacial muscle progenitor cells respond differently to elevated and reduced RA signaling.

**FIGURE 5:**
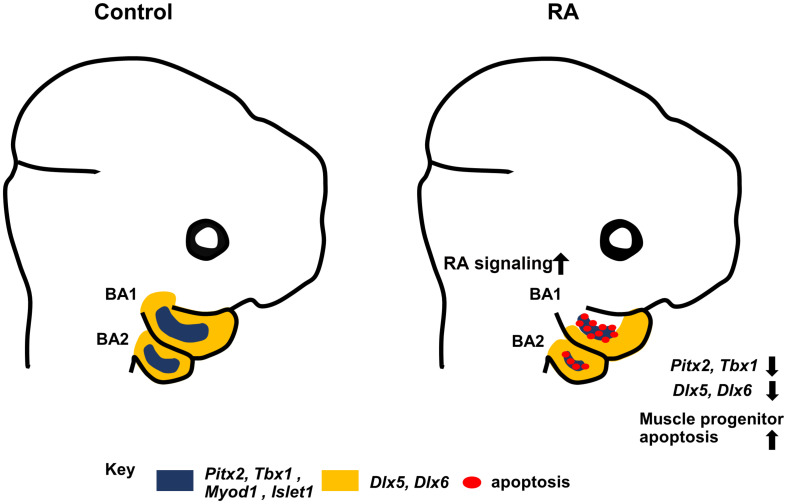
Summary diagram. Excess RA signaling results in disrupted expression of *Pitx2, Tbx1*, *MyoD1, and Islet1* (blue) in myogenic progenitors, and *Dlx5/6* (yellow) in CNCCs, together with elevated muscle progenitor apoptosis, which contributes to branchiomeric muscle defects in BA1 and BA2.

*Pitx2* mouse mutants exhibit a failure of BA1-derived muscle development in association with altered *MyoD* expression and elevated apoptosis of undifferentiated muscle progenitor cells (Dong et al., 2006). RA signaling has also been implicated in directly regulating *Pitx2* in embryonic eye development (Kumar and Duester, 2010) and it has been also shown that RA signaling and *Pitx2* regulate CNCCs during zebrafish development (Chawla et al., 2016). Exogenous RA (bead implantation) in chick embryos impacts the expression of the myogenic markers *Pitx2* and *Tbx1* (Bothe et al., 2011). Furthermore, Retinoid X receptor (RXR) co-localizes with PITX2 in extraocular muscle cells (Hebert et al., 2017). Together with our results, this clearly shows that perturbed RA signaling results in downregulation of *Pitx2* in BA1 and BA2, which in turn results in branchiomeric muscle defects. However, although muscles derived from BA1 are absent in *Pitx2* mutants, the muscles derived from BA2 are merely distorted (Shih et al., 2007). To explain the severe defects in muscles derived from BA2 in our study in association with elevated RA signaling, we examined the premyoblast specification marker, *Tbx1*. *Tbx1* mutants exhibit severe perturbation or absence of both BA1 and BA2 derived muscles (Kelly et al., 2004). Furthermore, *Pitx2* and *Tbx1* are known to be molecular partners that regulate parallel pathways of common target genes in craniofacial muscle development (Dong et al., 2006; Bothe et al., 2011; Braun and Gautel, 2011). Additionally, interactions between *Tbx1* and RA signaling have been demonstrated in a mouse model of DiGeorge syndrome (Ryckebusch et al., 2010). Consistent with these findings, we observed a substantial reduction of *Tbx1* expression in the mesodermal core of BA1 and BA2 in RA-treated embryos. Taken together, our results further support the conclusion that elevated RA signaling results in defects in the specification of undifferentiated muscle progenitor cells in BA1 and BA2, and thus contributes to the etiology of branchiomeric muscle malformation (Figure 5).

### RA-Induced Proximal Loss of *Dlx5/6* Impacts Branchiomeric Muscle Development

Previous *in vitro* and *in vivo* studies showed that proper RA signaling is essential for normal NCC development (Lee et al., 1995; Niederreither et al., 2003; Wang et al., 2019). Furthermore, CNCCs surround mesoderm cells in BAs that give rise to myogenic progenitors (Trainor et al., 1994; Trainor and Tam, 1995), and these cellular interactions are crucial for branchiomeric muscle formation (Noden, 1983, 1986; Chai et al., 2000; Sambasivan et al., 2011). For instance, *Dlx5*-positive CNCCs have been reported to guide the migration of muscle progenitors derived from BAs (Sugii et al., 2017). Additionally, CNCCs induce cranial myogenic formation by secreting inhibitory factors of the BMP and WNT signaling pathways in the BAs of chick embryos (Tzahor et al., 2003). In the present study, *Dlx5/6* expression was decreased in the proximal region of BA1 and BA2. The loss of *Dlx5/6* is known to impact muscle patterning and differentiation during the later stages of branchiomeric muscle development, however, Dlx5/6 do not affect the expression of the myogenic specification markers (Heude et al., 2010). Taken together, these facts suggest that several mechanisms contribute to the branchiomeric muscle defects observed in the present study. Firstly, early branchiomeric muscle formation is inhibited by defects in CNCC development (Heude et al., 2010) and subsequent differentiation and patterning are affected by altered *Dlx5/6* expression in BAs as a result of disturbed RA signaling. Failure of *Dlx5/6* expression leads to intrinsic tongue and sublingual muscle defects and masticatory muscle defects (Heude et al., 2010). Interestingly, in our study, reduced proximal *Dlx5/6* expression led to masticatory muscle progenitor cell defects, but intrinsic tongue and mylohyoid muscles, which derive from a different mesodermal source, were present. We speculate that elevated RA has a small effect on mesodermal precursors of those muscles at the stages and under the conditions we have investigated. Additionally, since muscle attachments to bone are critical for shaping bone during development, it is possible that absence of the muscles of mastication is one reason for retarded condylar and angular processes development in the mandibles RA-treated embryos.

### Expression of *Retinoic Acid Receptors* in Developing Head With Disturbed RA Signaling

*Retinoic acid receptors* mediate RA signaling and thus play important roles in transducing RA signaling during embryonic development (Rhinn and Dolle, 2012). In the present study, we detected subtle differences in the expression of most *RARs* and *RXRs* between E11.0 control and RA-treated embryos, with the exception of *RXRa*, which showed reduced expression in the RA-treated group. These results indicated that daily administration of 25 mg/kg RA from E8.5 to E10.5 does not critically influence the expression of either *RARs* or *RXRs* during craniofacial development. In contrast, we detected a substantial diminishment in retinoic acid receptor expression in embryos in which RA signaling was reduced. There is a large degree of functional redundancy between retinoic acid receptors (Mark et al., 2009), hence, further investigations including genetic complementation experiments will be required to reveal the effects of altered expression of retinoic acid receptors on craniofacial muscle development.

## Materials and Methods

### Animals and RA Administration

Pregnant female Institute of Cancer Research (ICR) mice (CLEA, Japan) were administered all-trans RA (25 mg/kg body weight) (Sigma-Aldrich) by oral gavage. All of the mice were housed with a 12 h dark-light cycle in which the light phase started from 8 a.m. RA (25 mg/ml in dimethylsulfoxide) was diluted 1/10 in corn oil just before use. Control animals were given the equivalent volume of the carrier. Oral gavage was performed once per day at gestation stages (from E8.5 to E10.5). Embryonic stage E0.5 was defined on the morning of vaginal plug confirmation. The approximate somite number in ICR mice at E8.5 was seven pairs.

*Rdh10*^*flox/flox*^ and *Cre-ERT2* mice were maintained and used as previously described (Sandell et al., 2007, 2012; Kurosaka et al., 2017). In order to eliminate *RDH10* from developing embryos, *Rdh10*^*flox/flox*^ female mice were crossed with *Cre-ERT2:Rdh10^*flox/flox*^* male mice followed by administration of tamoxifen at E7.0 as previously reported (Kurosaka et al., 2017). *Cre-ERT2* mice carry the gene for Cre recombinase fused to the estrogen receptor T2 cassette inserted into the Rosa 26 locus (Ventura et al., 2007). Consequently, recombination takes place in a ubiquitous manner after administration of tamoxifen (Hayashi and McMahon, 2002). *Rdh10*^*flox/flox*^ embryos were used as control samples throughout the study.

All animal experiments were performed in accordance with the guidelines of the Animal Care and Use Committee of the Osaka University Graduate School of Dentistry, Osaka, Japan. The committee on the ethics of animal experiments of the same university approved the study protocol (permit number: 26-028-0, 26-020-0).

### Micro-CT

We used the R_ mCT2 system (Rigaku) with scanning parameters 50 kV, 200 μA to perform micro-CT. Scans were reconstructed and analyzed using 3D viewer and Volume Rendering Control software (Rigaku), according to standardized protocols.

### Bone and Cartilage Staining

E18.5 embryos were skinned and eviscerated. The embryos were fixed in 100% ethanol overnight and then stained for 24 h with Alcian Blue (150 μg/ml in 20 ml of glacial acetic acid and 80 ml of 95% ethanol). After washing in 100% ethanol, soft tissues were dissolved in 2% KOH overnight and stained with Alizarin Red (50 μg/ml in 1% KOH) overnight. Stained embryos were kept in 20% glycerol/1% KOH until skeletons became clearly visible. Embryos were stored in 50% glycerol/50% water.

### *In situ* Hybridization

Whole-mount and sectional *in situ* hybridization was performed as described with minor modifications using digoxigenin (DIG)-UTP (Roche)-labeled antisense RNA probes corresponding to the sequences of *Myogenin*, *Pitx2*, *Tbx1*, *Myod1*, *Dlx5/6*, *Aldh1a2*, *Aldh1a3, Rara*, *Rarb*, *Rarg*, *Rxra, Rxrb*, and *Rxrg*. Sequences used were previously reported in the Allen Brain Atlas^1^. For all *in situ* hybridization analyses, a minimum of three embryos of each sample were examined per probe.

### Analysis of Apoptosis and Cell Proliferation

Analyses of apoptotic cells were performed using an *in situ* cell death detection kit (Roche) following the manufacturer’s instructions. For analyses of proliferation, samples were incubated with a mouse anti-pHH3 antibody (1:200, Millipore) at 4°C overnight followed by secondary Alexa-Fluor-488 donkey anti-mouse IgG (1:200, Invitrogen) for 6 h at room temperature for sections and overnight at 4°C for whole embryos. To label muscle progenitor cells in BA1 and BA2, sections were counterstained with a goat anti-Islet1 antibody (5 μg/ml, Abcam) at 4°C overnight, followed by secondary antibody (Alexa-Fluor-546 donkey anti-goat IgG, 1:200, Molecular Probes). Cells in at least five adjacent sections were counted in each assay. Statistical significance was assessed using Fisher’s exact test.

## Data Availability Statement

The raw data supporting the conclusions of this article will be made available by the authors, without undue reservation.

## Ethics Statement

The animal study was reviewed and approved by Animal Care and Use Committee of the Osaka University Graduate School of Dentistry.

## Author Contributions

QW and HK: conceptualization. QW, LX, MS, YU, LS, PT, HK, JM, and TY: methodology, resources, and writing-review and editing. QW, HK, and TY: validation and writing original draft. QW, HK and JM: formal analysis and investigation. HK, YU, and TY: funding acquisition. All authors contributed to the article and approved the submitted version.

## Conflict of Interest

The authors declare that the research was conducted in the absence of any commercial or financial relationships that could be construed as a potential conflict of interest.

## Footnotes

1 http://portal.brain-map.org/

## Supplementary Material

Supplementary Figure 1 Transmission electron microscope (TEM) image of intrinsic tongue muscles. TEM images of control **(A)** and RA treated **(B)** intrinsic tongue muscle at E13.5. Scale bars; 500 nm. MF, muscle fiber.

Supplementary Figure 2 *In situ* hybridization of *Myogenin* in E13.0 head. *Myogenin in situ* hybridization for whole-mount in E13.0 Control (left) and *Cre- ERT2:Rdh10^*flox/flox*^* (Right) head. au, auricularis muscle; bu, buccinators muscle; ma, masseter muscle; te, temporalis muscle; zy, zygomaticus muscle.

Supplementary Figure 3 *In situ* hybridization of *Aldh1a2* and *Aldh1a3* in E10.5 branchial arches. Whole mount *in situ* hybridization of *Aldh1a2* and *Aldh1a3* in E10.5 RA-treated and *Cre Ert2;Rdh10^*flox/flox*^* embryos. The labels on the left indicate the genes whose expression was examined and the labels on the top indicate the condition of the sample treatments.

Supplementary Figure 4 *In situ* hybridization of *Retinoic acid receptors* (*Rars* and *Rxrs*) in E10.5 branchial arches. Whole mount *in situ* hybridization of *Rara*, *Rarb*, *Rarg*, *Rxra, Rxrb*, and *Rxrg* in RA-treated and *CreErt2;Rdh10^*flox/flox*^* embryos. Labels on the left indicate the genes whose expression was examined and labels on the top indicate the condition of the sample treatments. Asterisks indicate the position where different expression profiles could be detected among comparable samples.
