# Supplementary figures and images for: Branchiomeric Muscle Development Requires Proper Retinoic Acid Signaling
Source: Front Cell Dev Biol. 2021 Jul 9;9:596838. doi: 10.3389/fcell.2021.596838 (PMC8299418; doi:10.3389/fcell.2021.596838)

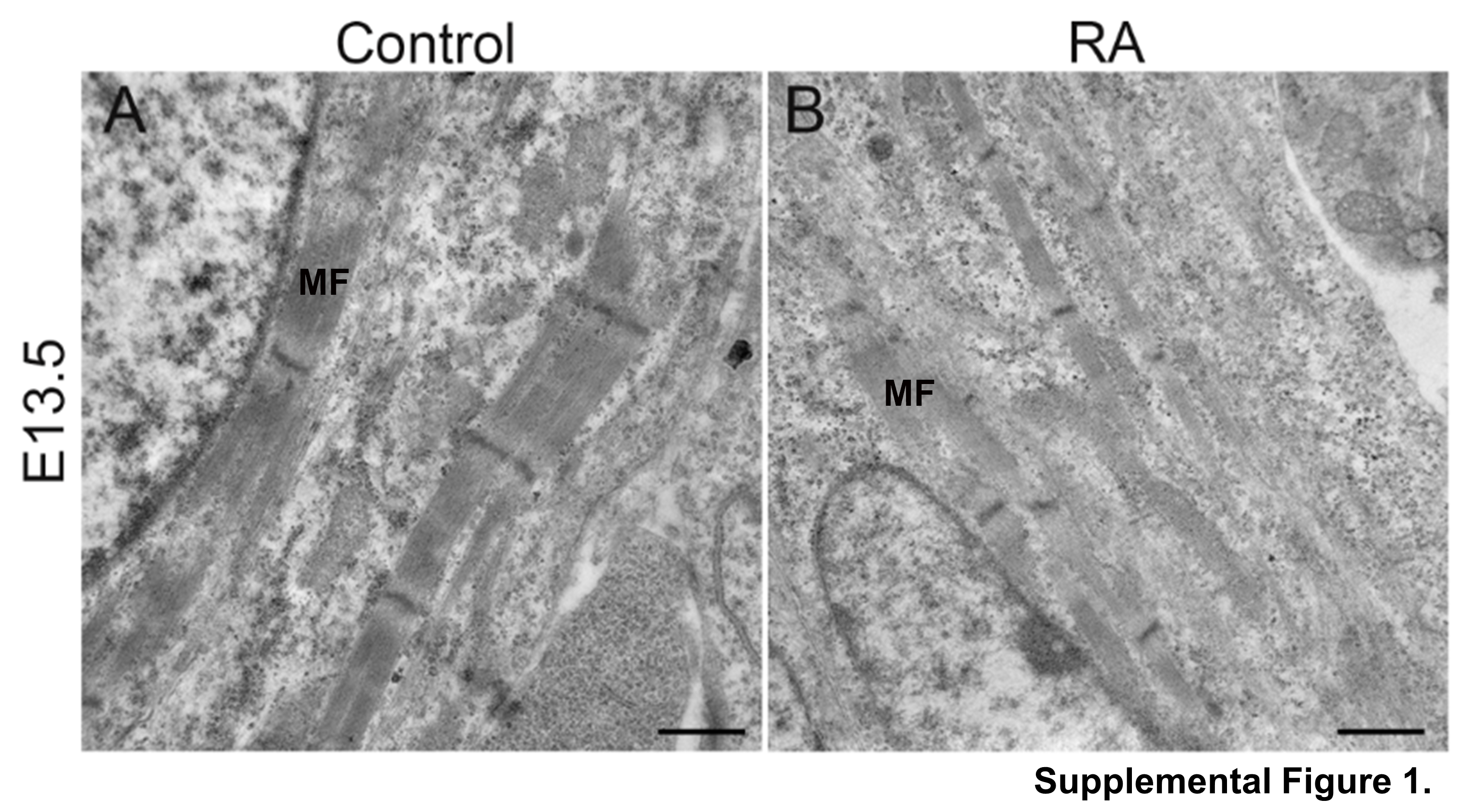

Supplement: Supplementary Figure 1 — Transmission electron microscope (TEM) image of intrinsic tongue muscles. TEM images of control (A) and RA treated (B) intrinsic tongue muscle at E13.5. Scale bars; 500 nm. MF, muscle fiber. [file Image_1.tif]

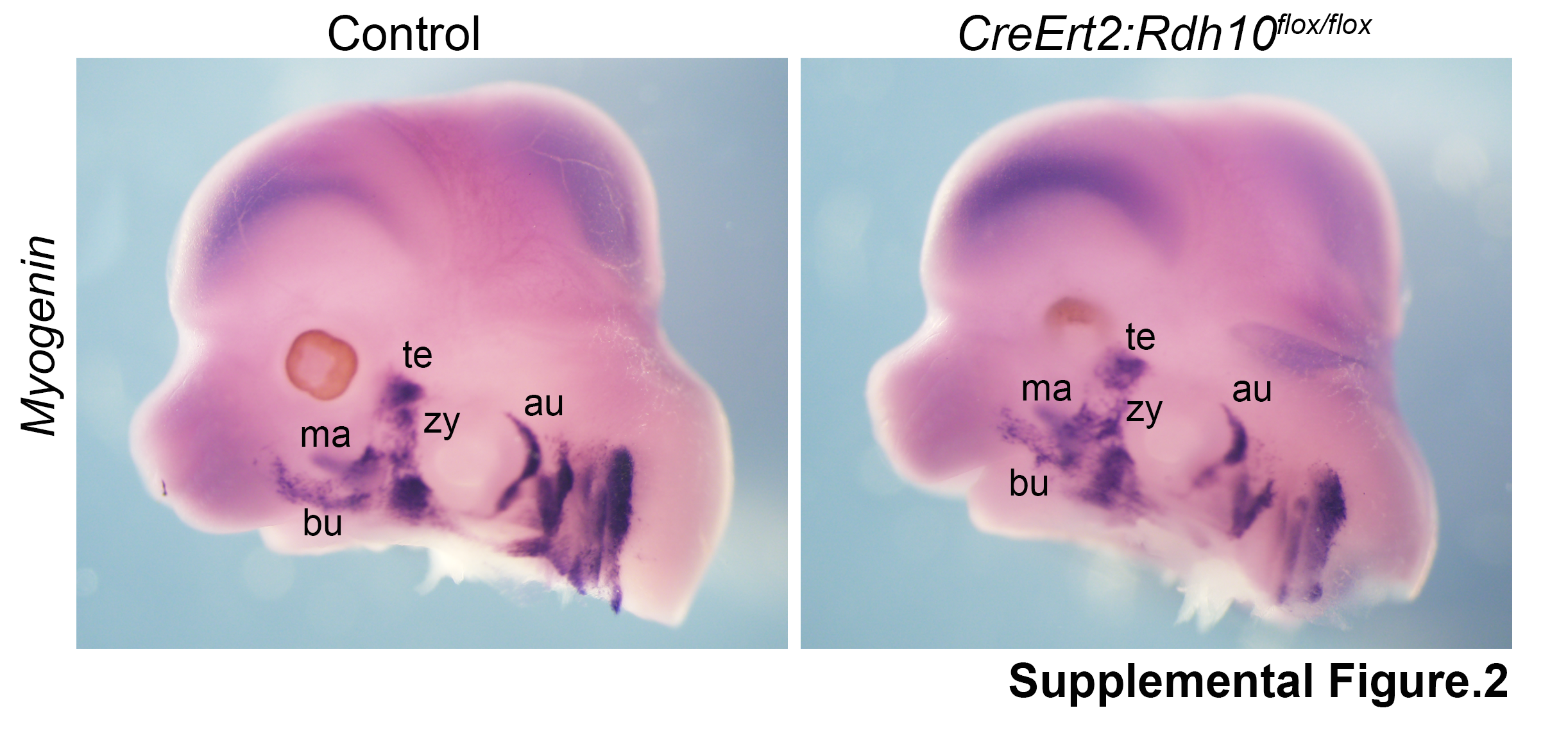

Supplement: Supplementary Figure 2 — In situ hybridization of Myogenin in E13.0 head. Myogenin in situ hybridization for whole-mount in E13.0 Control (left) and Cre- ERT2:Rdh10flox/flox (Right) head. au, auricularis muscle; bu, buccinators muscle; ma, masseter muscle; te, temporalis muscle; zy, zygomaticus muscle. [file Image_2.tif]

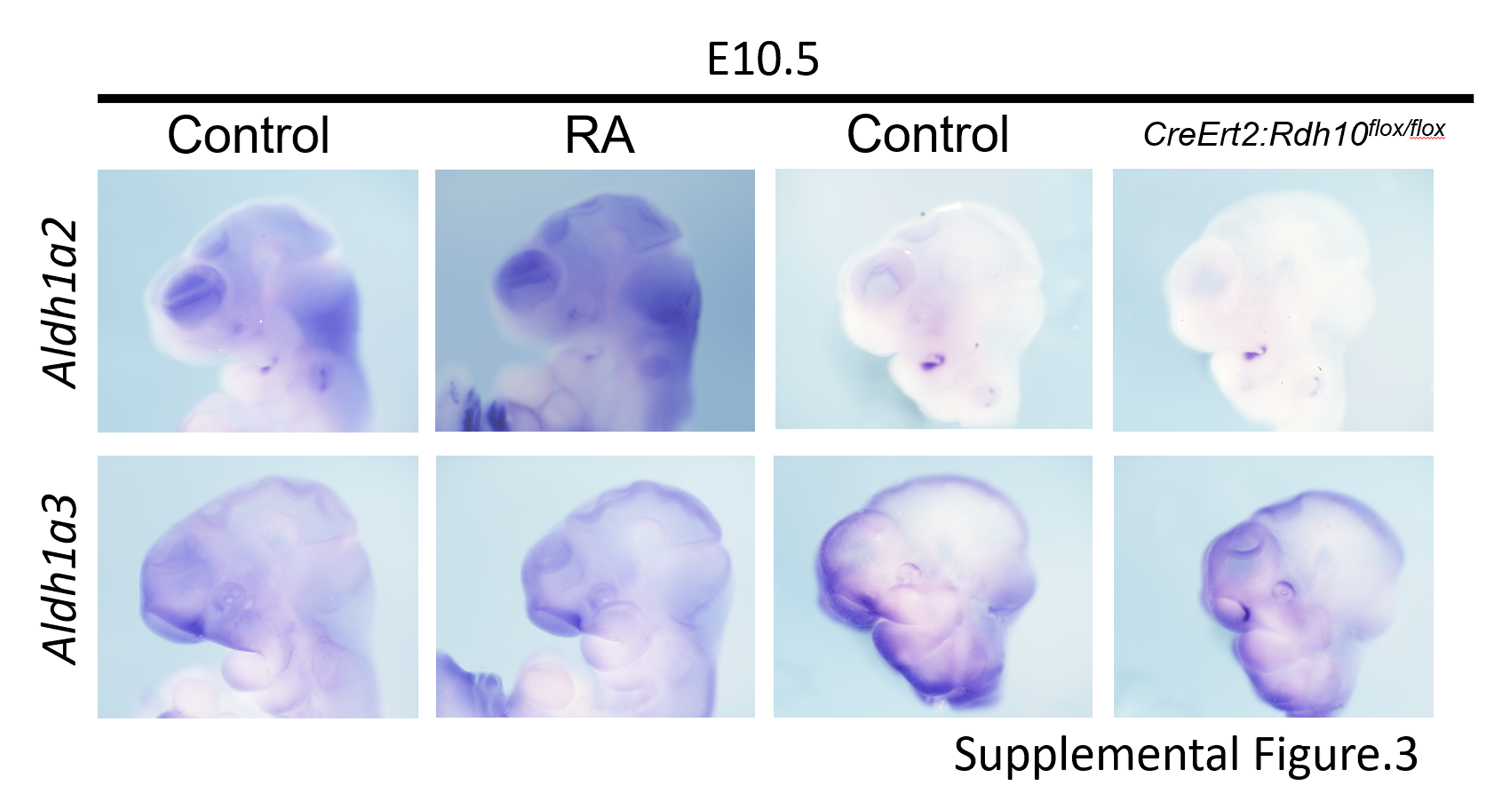

Supplement: Supplementary Figure 3 — In situ hybridization of Aldh1a2 and Aldh1a3 in E10.5 branchial arches. Whole mount in situ hybridization of Aldh1a2 and Aldh1a3 in E10.5 RA-treated and Cre Ert2;Rdh10flox/flox embryos. The labels on the left indicate the genes whose expression was examined and the labels on the top indicate the condition of the sample treatments. [file Image_3.tif]

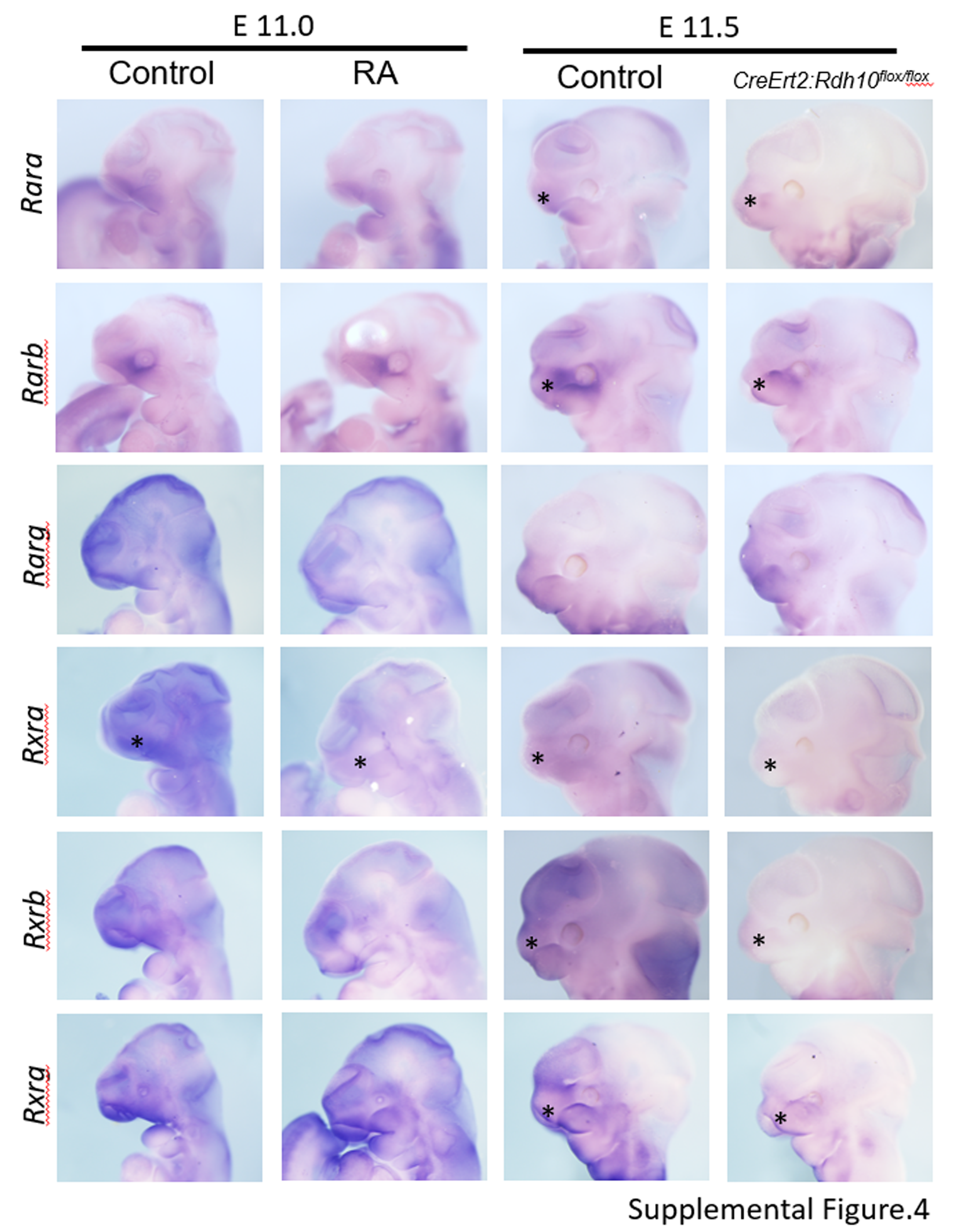

Supplement: Supplementary Figure 4 — In situ hybridization of Retinoic acid receptors (Rars and Rxrs) in E10.5 branchial arches. Whole mount in situ hybridization of Rara, Rarb, Rarg, Rxra, Rxrb, and Rxrg in RA-treated and CreErt2;Rdh10flox/flox embryos. Labels on the left indicate the genes whose expression was examined and labels on the top indicate the condition of the sample treatments. Asterisks indicate the position where different expression profiles could be detected among comparable samples. [file Image_4.tif]
